# Effectiveness of visual inspection compared with non-microbiologic methods to determine the thoroughness of post-discharge cleaning

**DOI:** 10.1186/2047-2994-2-26

**Published:** 2013-10-02

**Authors:** Graham M Snyder, Aleah D Holyoak, Katharine E Leary, Bernadette F Sullivan, Roger B Davis, Sharon B Wright

**Affiliations:** 1 Division of Infectious Diseases, Department of Medicine, Beth Israel Deaconess Medical Center, Boston, MA 02215, USA; 2 Division of Infection Control/Hospital Epidemiology, Silverman Institute of Health Care Quality & Safety, Beth Israel Deaconess Medical Center, 330 Brookline Avenue, Mailstop SL-435, Boston, MA 02215, USA; 3 Department of Medicine, Division of General Medicine and Primary Care, Beth Israel Deaconess Medical Center, Boston, MA 02115, USA

**Keywords:** Environmental cleaning, Surface contamination, Nosocomial transmission

## Abstract

**Background:**

Published data to date have provided a limited comparison between non-microbiologic methods—particularly visual inspection—and a microbiologic comparator to evaluate the effectiveness of environmental cleaning of patient rooms. We sought to compare the accuracy of visual inspection with other non-microbiologic methods of assessing the effectiveness of post-discharge cleaning (PDC).

**Methods:**

Prospective evaluation to determine the effectiveness of PDC in comparison to a microbiologic comparator. Using a highly standardized methodology examining 15 high-touch surfaces, the effectiveness of PDC was evaluated by visual inspection, the removal of fluorescent marker (FM) placed prior to room occupancy, quantification of adenosine triphosphate (ATP) levels, and culture for aerobic colony counts (ACC).

**Results:**

Twenty rooms including 293 surfaces were sampled in the study, including 290 surfaces sampled by all four methods. ACC demonstrated 72% of surfaces to be microbiologically clean. Visual inspection, FM, ATP demonstrated 57%, 49%, and 66% of surfaces to be clean. Using ACC as a microbiologic comparator, the sensitivity of visual inspection, FM, and ATP to detect a clean surface were 60%, 51%, and 70%, respectively; the specificity of visual inspection, FM, and ATP were 52%, 56%, and 44%.

**Conclusions:**

In assessing the effectiveness of PDC, there was poor correlation between the two most frequently studied commercial methods and a microbiologic comparator. Visual inspection performed at least as well as commercial methods, directly addresses patient perception of cleanliness, and is economical to implement.

## Background

The U.S. Centers for Disease Control and Prevention, in the publication *Guidelines for Environmental Infection Control in Healthcare Facilities*, recommends environmental sampling in the setting of an outbreak investigation, as part of research investigating healthcare-associated infections, for the monitoring of potential environmental hazards, and for quality assurance [1]. These recommendations emphasize rigorous methodologies to ensure accuracy of results. Further guidelines on the monitoring of environmental cleaning of patient rooms, including post-discharge cleaning (PDC), outline the advantages and limitations of five available methods: direct observation of cleaning practice (such as visual inspection), cultures of surfaces obtained by swab, cultures of surfaces obtained by Agar slide, detection after cleaning of a fluorescent marker (FM) placed prior to cleaning, and sampling for the presence of adenosine triphosphate (ATP) as a marker for organic material [2,3]. While they do not directly assess for the presence of pathogens, visual inspection, FM and ATP methods are easy to implement; unlike microbiologic techniques, they allow for evaluation of effectiveness immediately after PDC is performed. It has been demonstrated that the quality of environmental cleaning as measured by culture of pathogens may be improved through education of environmental services staff [4].

To date, multiple studies have investigated the accuracy of ATP quantification to assess the effectiveness of environmental cleaning, compared to either visual inspection or microbiologic evaluation of cleanliness [5-12]. These studies do not, however, make a direct analytic comparison of visual inspection to a microbiologic comparator for the purposes of determining the accuracy of visual inspection in determining the effectiveness of PDC. One study has described both FM and microbiologic methods of evaluating an educational intervention to improve cleaning practices [13], and multiple studies have described the use of a FM to evaluate environmental cleaning [14-20], but without a statistically rigorous comparison of methods. A recent study investigating the efficacy of PDC on five high-touch surfaces quantified aerobic colony count (ACC) and ATP immediately before and after PDC, as well as removal of FM placed immediately before PDC [21].

In this prospective study, we sought to evaluate quantitatively the test characteristics of visual inspection to determine the effectiveness of PDC in reference to a microbiologic comparator, and in comparison to FM and ATP methods.

## Methods

### Study design

This study was conducted from April through June 2011, at the Beth Israel Deaconess Medical Center (BIDMC), a 661-bed academic tertiary care hospital in Boston, Massachusetts, U.S.A. Bed availability and completion of PDC were monitored through the hospital electronic bed tracking system. A convenience sample of all unoccupied terminally-cleaned rooms available during weekday business hours were evaluated for this study. In these terminally-cleaned rooms prior to patient occupancy, pre-determined surfaces were tagged with a low-visibility FM. After patient discharge and prior to occupancy by the next patient, PDC was evaluated by assessing removal of FM, visual inspection, quantification of ATP, and microbiologic sampling. Environmental services staff were neither aware of nor participated in the conduct of this study.

During the study period, no changes were made in the hospital room cleaning or infection control practices. Standard practice for PDC at BIDMC includes the use of a one-step quaternary ammonium-based disinfectant cleaners (Virex II 256, Diversey) disinfectant, with the use of bleach-based disinfection only in exceptional circumstances. Environmental services staff are assigned to individual units on weekdays with coverage on weekends and holidays. Routine non-PDC occurs daily and is less rigorous than PDC practice.

This study was designed and implemented as a quality improvement initiative and approved as a research investigation by the institutional review board at BIDMC. Patients were not involved in the study, and no patient- or staff-identifying information was collected. The products used in the study were obtained through BIDMC, and the developers and manufacturers of the products used were not involved in the design, conduct, or publication of the study.

### Data collection

Five members of the Infection Control/Hospital Epidemiology division collected data for this study (including G.S., A.H., K.L. and B.S., all of whom were involved in the design of the study). For members who were not highly experienced in the field, training in the observation of room cleaning was conducted by both Infection Control/Hospital Epidemiology division members and environmental services staff, with a minimum of at least 5 room observations. Two trained observers jointly performed data collection for each PDC room observed, and resolved discrepancies in assessment by consensus. A kappa statistic was not calculated. Observers, room location, and dates and times relevant to study events were recorded. All data were recorded in a relational database (Access 2003, Microsoft).

We selected for analysis 15 of the 17 common high-touch surfaces recommended by the CDC for sampling, including surfaces in both the patient room and adjoining bathroom [2]. Visual inspection of the entire surface was performed after PDC and at the time of FM assessment. ATP and microbiologic sampling were performed immediately after performing FM and visual inspection evaluations, and each was performed in a non-overlapping area immediately adjacent to the FM. For objects with an irregular (non-flat) surface, a standardized area was sampled for the ATP and microbiologic methods. The surfaces sampled and the standardized sampling techniques are described in Table 1.

**Table 1:** Surfaces and sampling technique for the comparison of methods to assess thoroughness of environmental cleaning

	**Sampling method**	
**Location**	**Fluorescent marker**	**Adenosine triphosphate and microbiologic sampling**
Bed rail	Between raised control buttons on the outer surface of the railing closest to room door	Adjacent to fluorescent marker
Overbed tray table	On the upper surface of the table, adjacent to and centered on the edge of the table closest to the hand-operated height adjustment mechanism	Adjacent to fluorescent marker
Call button	On the hand-held device, centered between the emergency button and speaker	Adjacent to fluorescent marker, including raised control buttons
Bedside telephone	Top center of the surface of the receiver, on the obverse side of the earpiece	Adjacent to the fluorescent marker including lateral dial surfaces
Bedside table	Corner closest to the patient bed on the drawer-side (front) top surface [if more than one table is present in the room, the table located to the patient’s right]	Adjacent to fluorescent marker
Chair	On the surface of the seat, in the rear corner closest to the patient bed [if more than one chair present in the room, the chair located closest to the patient bed]	Adjacent to fluorescent marker
Room sink	Corner of the surface adjacent to the basin, closest to the wall and room door	Adjacent to fluorescent marker
Room light switch	Upper left corner of the wall plate	Surface of the light switch plate, over a 2×2-inch surface around but not touching the switch itself
Room door knob, inner	Upper middle of the door plate behind the handle	Surface of the door plate, over a 2×2-inch surface around but not touching the handle
Bathroom light switch	Upper left corner of the wall plate [if electrical sockets are present in upper left, then the upper right corner]	Surface of the light switch pad, over a 2×2-inch surface around but not touching the switch itself
Bathroom hand rail (adjacent to toilet)	Lateral surface of the portion of the handrail farthest from the toilet	Adjacent to the fluorescent mark, with a circumferential sampling of a 2-inch length of railing
Bathroom sink	Corner of the surface adjacent to the basin, to the left and rear of the sink surface	Adjacent to fluorescent marker
Toilet seat	Rear left corner of the seat on the upper surface	Adjacent to fluorescent marker
Toilet flush handle	Superior surface of the handle closest to the plumbing connection	Adjacent to the fluorescent marker, with a circumferential sampling of 1/2 of the handle length
Bedpan cleaner	Surface of the cleaning mechanism behind the spray head (excluding the handle)	Adjacent to fluorescent marker, sampling all surfaces of a lateral half of the cleaning mechanism for a 2-inch length

### Sampling methods

In a cleaned room prior to patient admission, approximately 1 mL of fluorescent gel was applied to specified surfaces in a 1-cm diameter circle (Glo Germ Gel, Glo Germ and DigiGlo, EcoLab). FM was assessed with an ultraviolet light following patient discharge and PDC. The absence of fluorescence was defined as a clean surface while a fully intact or a partially removed mark was defined as dirty. There is no established standard for placing FM prior to room occupancy or immediately prior to PDC; prior studies have been conducted using both time points [13,15,21,22], and a patent for a commonly used FM product states that evaluation may take place “after a single cleaning opportunity or multiple cleaning opportunities” [23]. In order to ensure blinding of environmental services staff to the cleaning assessment, FM were placed following previous PDC and prior to patient occupancy.

Visual inspection was performed at the time of FM evaluation. The entire surface was inspected for the presence of four discrete pre-specified contaminants—dust, tape/tape residue, hair, and moisture—as well as a category for any other contaminants identified. The surface was classified as dirty if visual inspection demonstrated one or more of the five contaminant types.

ATP assessment of environmental cleaning was performed contemporaneously with assessment of FM, using the 3 M Clean-Trace Surface ATP System (3 M). Consistent with product directions, the pre-moistened manufacturer-supplied swab was rubbed over an approximately 2 × 2 inch area, first covering the area with a back-and-forth pattern and subsequently in an overlapping but perpendicular back-and-forth pattern, performed with a twisting motion to expose the entire swab to the surface. Samples were analyzed promptly after collection and according to the manufacturer’s directions. Using the 3 M Clean-Trace NG Luminometer (3 M), the ATP present was quantified as relative light units (RLU). A clean surface was defined as one with a measured RLU > 250, consistent with prior studies [8,24].

Microbiologic sampling was performed using two sterile cotton-tipped swabs moistened with sterile water rubbed simultaneously in a manner identical to that used for ATP sampling. One swab each was then used to inoculate a 100 mm Trypticase^TM^ soy agar with 5% sheep blood and a mannitol salt agar plate (Becton Dickinson). Both plates were incubated aerobically at 37° Celsius. After 24 hours of incubation time, the total number of colonies on sheep blood agar were counted (aerobic colony count, ACC). Mannitol salt agar plates were examined after 48 hours of incubation for the presence of *Staphylococcus aureus*, which was inferred by the growth of characteristic colonies on both sheep blood and mannitol salt agar and the presence of fermentation as indicated by pink-to-yellow color change on mannitol salt agar plates. *A priori*, we decided that up to 5 colony-forming units (CFU) of aerobic flora would be an acceptable limit of contamination for the standard surface we sampled based on the available literature and the methodology used.

### Statistical analysis

We compared the three non-microbiologic methods to assess effectiveness of PDC to each other and to a microbiologic comparator through three analyses: the thoroughness of disinfection cleaning (TDC) score, the concordance of clean/dirty test results with the microbiologic comparator, and test characteristics compared with the microbiologic comparator.

The TDC score for each method was calculated as the percentage of evaluated surfaces determined to be clean [3]. Surfaces characterized as either clean or dirty by a non-microbiologic method and the microbiologic comparator (clean/clean or dirty/dirty) were considered concordant results, and surfaces for which there was disagreement between the two methods (clean/dirty or dirty/clean) were considered discordant. Percent discordance between two tests was calculated as the fraction of total paired observations for which the two methods were discordant among all paired observations. Sensitivity, specificity, positive predictive value, and negative predictive value for visual inspection, FM, ATP were calculated relative to the ACC comparator in the standard fashion [25].

The null hypothesis that all three non-microbiologic methods demonstrated the same TDC scores was tested using a chi-square test. Statistical tests of the primary analyses used a two-sided 0.05 level of significance; a Bonferroni correction was applied for pairwise comparisons of non-microbiologic methods. Statistical analyses were performed using STATA software (version 10.0, Stata Corp).

## Results

A total of 20 of 50 (40.0%) candidate rooms (marked with FM) were evaluated after PDC. The remaining FM-marked rooms were not evaluable due to rapid admission of a patient after PDC. A total of 293 surfaces in these 20 rooms were sampled by one or more methods in this study, and 290 (99.0%) were sampled by all 4 methods. Of the three surfaces sampled not included in the analysis, two surfaces in one room could not be evaluated due to interruption by patient admission, and for one surface ACC was not adequately obtained.

Among the 290 surfaces tested by all four methods, 72.1% (209) were microbiologically clean with ACC ≤ 5 CFU. A total of 107 (36.9%) surfaces demonstrated no growth on sheep blood agar. Inferred *S. aureus* growth was identified on 41 (14.1%) of surfaces sampled. Visual inspection demonstrated one or more elements of contamination on 125 (43.1%) surfaces, including: dust (56), tape (31), hair (15), moisture (8), and other (50). Contamination classified as “other” included stain(s) (20), debris (17), and sticky substance, soap residue, grime, removable mark(s), toothpaste, dirt, lint, tissue, or fingerprints in five or fewer instances. The mean ACC, percent clean by visual inspection and FM, and median ATP RLU measurement for each surface type sampled is described in Table 2.

**Table 2:** Effectiveness of post-discharge cleaning of high-touch surfaces, evaluated by four methods

**Surface sampled**	**Number sampled**	**Mean aerobic colony count (± SE)**	**Number (%) clean by visual inspection**	**Median ATP RLU (range)**	**Number (%) clean by FM**
Bedrail	20	1.5 (0.9)	9 (45.0)	63 (13–806)	6 (30.0)
Tray table	20	2.3 (1.2)	11 (55.0)	123.5 (26–4185)	17 (85.0)
Call button	20	38.7 (25.8)	10 (50.0)	276 (23–3601)	15 (75.0)
Telephone	20	7.7 (2.5)	12 (60.0)	166 (30–1863)	16 (80.0)
Bedside table	20	1.9 (0.9)	9 (45.0)	91 (15–889)	9 (45.0)
Chair	16	23.9 (13.1)	9 (56.3)	305.5 (53–1472)	3 (18.8)
Room sink	16	9.1 (3.7)	6 (37.5)	94.5 (11–511)	8 (50.0)
Room light switch	19	5.5 (2.2)	15 (79.0)	49 (5–314)	6 (31.6)
Room door knob	20	5.7 (2.9)	10 (50.0)	108.5 (18–354)	2 (10.0)
Bathroom light switch	20	4.4 (2.1)	13 (65.0)	138.5 (15–1716)	5 (25.0)
Bathroom hand rail	20	204.7 (195.6)	11 (55.0)	284 (14–6068)	5 (25.0)
Bathroom sink	20	10.5 (6.2)	7 (35.0)	160 (30–1610)	13 (65.0)
Toilet seat	20	19.5 (15.1)	18 (90.0)	74.5 (14–258)	19 (95.0)
Toilet flush handle	19	133.8 (78.5)	16 (84.2)	179 (33–1245)	14 (73.7)
Bedpan cleaner	20	21.4 (9.8)	9 (45.0)	190.5 (10–1530)	5 (25.0)
Total	290	32.9 (14.7)	165 (56.9)	130.5 (10–6068)	143 (49.3)

Note: SE, standard error; ATP, adenosine triphosphate; RLU, relative light units; FM, fluorescent marker.

The TDC scores for each of the three non-microbiologic methods tested were (n=290): visual inspection 56.9%, FM 49.3%, ATP 66.2% (cutoff, RLU > 250) (Table 3). The TDC scores for these three methods were not statistically the same (p=0.002). The TDC scores for visual inspection and FM, and visual inspection and ATP were not statistically different (p=0.20 and p=0.06, respectively) although the TDC scores between FM and ATP were significantly different (p>0.001).

**Table 3:** Test characteristics for three methods of determining effectiveness of post-discharge cleaning as tested against a microbiologic comparator

		**Test characteristics to determine clean (95% CI)**			
**Test**	**TDC score**	**Sensitivity**	**Specificity**	**Positive predictive value**	**Negative predictive value**
ACC ≤ 5 CFU	72.1%	—	—	—	—
Fluorescent marker	49.3%	51.2% (44.2-58.2)	55.6% (44.1-66.6)	74.8%	30.6%
Visual inspection	56.9%	60.3% (53.3-67.0)	51.9% (40.5-63.1)	76.4%	33.6%
ATP (RLU > 250)	66.2%	70.3% (63.6-76.4)	44.4% (33.4-55.9)	76.6%	36.7%

Note. CI, confidence interval; ACC, aerobic colony count; CFU, colony-forming units; TDC, thoroughness of disinfection cleaning; ATP, adenosine triphosphate; RLU, relative light units.

The test characteristics of the three methods tested compared with the microbiologic comparator are demonstrated in Table 3. The sensitivity of FM, visual inspection, and ATP methods to detect a surface with low microbial contamination were 51.2%, 60.3%, and 70.3%, respectively. The specificity of all three non-microbiologic methods was less than 60%. All three non-microbiologic methods demonstrated a positive predictive value of approximately 75% and a negative predictive value of between 30% and 37% (Table 3). Table 4 describes the concordance and discordance between the non-microbiologic methods and the microbiologic comparator. When compared with visual inspection, FM and ATP were discordant in 108 (37.2%) and 105 (36.2%) of 290 surfaces sampled and when compared with each other FM and ATP were discordant in 135 (46.6%) of 290 surfaces sampled.

**Table 4:** Concordance and discordance between non-microbiologic and microbiologic methods to determine the effectiveness of post-discharge cleaning

	**Surfaces concordant and discordant with microbiologic sampling (CFU ≤ 5)**			
	**Concordant, clean**	**Concordant, dirty**	**Discordant clean/dirty**^**†**^	**Discordant dirty/clean**^**‡**^
	**N**	**N**	**N (%)**	**N (%)**
Fluorescent marker	107	45	102 (35.2)	36 (12.4)
Visual inspection	126	42	83 (28.6)	39 (13.5)
Adenosine triphosphate	147	36	62 (21.4)	45 (15.5)

Note: A total of 290 surfaces were sampled; 209 (72.1%) were microbiologically clean.

†Discordant clean/dirty indicates the microbiologic method characterized the surface as clean, and the non-microbiologic method characterized the surface as dirty.

‡Discordant dirty/clean indicates the microbiologic method characterized the surface as dirty, and the non-microbiologic method characterized the surface as clean. CFU, colony-forming units.

## Discussion

In this study, we found that the performance of visual inspection was comparable to two commonly used non-microbiologic methods of determining the effectiveness of PDC when compared to a microbiologic comparator. However, all three non-microbiologic methods demonstrated poor correlation with our microbiologic comparator and with each other. Visual inspection, FM, and ATP demonstrated findings discordant with microbiologic results in 42%, 48% and 37% of the surfaces tested.

These findings suggest that none of these three methods as implemented give a “true” estimate of the effectiveness of PDC when using ACC as the comparator. Each method is more likely to falsely determine a surface is dirty when there is low microbial contamination than they are to falsely report a surface as clean. This effect is most pronounced with the FM method. However, false negative and false positive results indicate different issues: any positive result for FM or ATP testing (including false positive results) indicates inadequate cleaning practices, while a false negative result for FM and ATP methods suggests a limitation of these non-microbiologic methods in assessing the reduction in the risk of transmission of bacterial pathogens.

Although it has been stated that comparisons between the FM and ATP methods may not be valid because they measure different properties of cleanliness (how well a surface is wiped clean in contrast to the quantity of organic material contaminating a surface), [26] both have been recommended to evaluate effectiveness of PDC [3]. While distinct in their properties, one limitation of both methods is similar—they do not directly quantify the presence of a microbial pathogen that may be transmitted between consecutive patients.

Prior infection control studies that have evaluated surface cleanliness using microbiologic techniques most commonly use contact plates for sampling [6,10,21,27]. These studies, as well as similar studies that use swab techniques, and proposed standards have defined a surface as clean when there is growth > 2.5 or 5.0 CFU/cm^2^[6,10,11,13,21,27-29]. However, these studies employ significantly heterogeneous methods. A contact plate method may be difficult to implement or require modulation of the technique for culturing of irregular surfaces [6,10,21,27,29]. Sampling with a swab technique has included an enrichment process which increases the cost and complexity of the evaluation [11,13,29]. We therefore used a method of microbiologic sampling of post-discharge cleaned surfaces that would predictably yield lower quantitative growth but is simple and inexpensive to employ, sampled regular and irregular surfaces in a uniform fashion, and was performed in the same fashion as ATP sampling.

When performed by a trained observer, visual inspection was comparably accurate to FM and ATP methods. Visual inspection “sampled” the entire surface, while in contrast, FM, ATP, and ACC sample a limited but highly standardized surface area. However, all three non-microbiologic methods were conducted in this study in a fashion consistent with their day-to-day practice. Quite importantly in regards to general hospital quality improvement, visual inspection is the only measure of cleanliness to address the aspect of cleanliness that is readily apparent to a patient. An additional advantage of visual inspection over FM and ATP methods is lower cost. While training requirements are of low complexity comparable to FM and ATP, personnel time requirements for visual inspection are similar to ATP and less than that for FM (which requires two room entries). However, ATP and FM methods also require ongoing material costs for each surface tested.

Prior studies have demonstrated improvement in PDC practices after implementation of the FM method [13,19]. While efforts were made in our study and others to blind environmental services staff to the intervention, it is possible that the improvement in PDC demonstrated in some studies is a result due in part to the Hawthorne effect, as has been demonstrated with other infection control interventions [30,31], rather than an effect specifically due to feedback resulting from FM findings. In our study, PDC assessment did not take place while the cleaning was being performed, nor in the presence of environmental services staff thus eliminating this issue. Similarly, we chose to place the marker prior to patient admission rather than immediately prior to room cleaning (as could be implemented by product specifications) when the Hawthorne effect would be theoretically more pronounced [23]. While generally colorless, in this study we did not find the FM to be entirely invisible; upon learning that this method is being used to assess effectiveness of PDC, environmental services staff may target cleaning efforts to the FM without improving overall cleaning effectiveness. The visual inspection and ATP methods performed after PDC are not likely to be susceptible to the Hawthorne effect.

A primary limitation of our study is the use of a pragmatic microbiologic “standard” to compare the effectiveness of PDC as assessed by non-microbiologic methods. A true standard among all currently available methods and among microbiologic methods to evaluate effectiveness of PDC has not been established. However, since the principal objective is to reduce the nosocomial transmission of pathogens via fomites by undertaking thorough cleaning practices, a microbiologic comparator would be appropriate and has been used in prior studies [10,12]. Our method of sampling was chosen for several reasons, including similarity in implementation to ATP sampling, relative ease of use, and generalizability. While this method will likely underreport microbial contamination and will not detect pathogens such as *Clostridium difficile* or viral pathogens that may also be transmitted nosocomially via fomites, a single method to ascertain the presence of all pathogens is not feasible. A pathogen-specific method, such as the identification of *S. aureus* in this study, would be unlikely to yield a sufficient number of positive samples from which to draw meaningful comparisons. To our knowledge, there is no reported comparison of direct plating and enrichment methods for environmental sampling.

In this study, there was a significant range of effectiveness of PDC. Thus, our findings may be less applicable to hospitals with a very narrow range of PDC effectiveness. We used a convenience sample of available rooms. However, since we performed observations in all units of the hospital, we believe the effect of non-random room sampling on the internal validity or generalizability of the study is likely small. We did not collect data on specific individuals performing PDC, and so cannot exclude an effect of individual practice patterns on the study results. However, sampling rooms throughout the medical center likely mitigates this potential effect. Furthermore, it has previously been determined that there is greater variability in the TDC score when comparing type of surface than when comparing patient unit [3]. While there are minor variations in the methodology used, our study demonstrated similar TDC scores and distribution of findings compared with prior studies: FM, 20-90% [13,19], and ATP, median RLU values approximately 100–500 and overall range from > 50 to < 13,000 [12].

One potential strategy to evaluate the effectiveness of PDC is to implement a two-tiered approach. For routine evaluation of the effectiveness of PDC, visual inspection may be used. While in our study and others visual inspection lacks good correlation with microbial contamination [8,10,11], we did not identify an appreciably more efficacious method of assessing PDC. Patient perception of hospital cleanliness is an increasingly important element of patient satisfaction [32], and visual inspection of PDC directly addresses this issue. Other studied methods may be more difficult to implement, and both FM and ATP methods would entail a higher cost than visual inspection. In the setting of a cluster of infections with a specific organism for which it is suspected that the environment may play a significant role in patient acquisition and transmission, culture- or polymerase chain reaction-based methods could be implemented to assess effectiveness of PDC to limit further nosocomial transmission of the specific organism [33,34].

In conclusion, we have found that three existing methods to determine the effectiveness of PDC significantly lack diagnostic precision when compared to a microbiologic comparator. Given this comparable limitation of all tested non-microbiologic methods, visual inspection performed in a standardized fashion may be a preferred method of assessing PDC given its additional advantages in addressing patient satisfaction and cost of implementation.

## Abbreviations

PDC: Post-discharge cleaning; FM: Fluorescent marker; ATP: Adenosine triphosphate; ACC: Aerobic colony counts; RLU: Relative light units; TDC: Thoroughness of disinfection cleaning (score); CFU: Colony-forming units.

## Competing interests

The authors declared that they have no competing interests.

## Authors’ contributions

GS participated in the design, conduct and analysis of the study, as well as manuscript preparation. AH participated in study design and conduct, and manuscript preparation. KL participated in the conduct of the study. BS participated in the study design and manuscript preparation. RD participated in study analysis and manuscript preparation. SW participated in the study design and analysis, and manuscript preparation. All authors have read and approved the final manuscript.
